# Clinical and Demographic Profile of Wilson Disease in Young Adults: A Retrospective Study at a Tertiary Care Center in Peshawar, Pakistan

**DOI:** 10.7759/cureus.100661

**Published:** 2026-01-03

**Authors:** Imran Khan, Mehwash Iftikhar, Sheraz J Khan

**Affiliations:** 1 Internal Medicine, Medical &amp; Teaching Institution Hayatabad Medical Complex, Peshawar, PAK; 2 Medicine, Medical &amp; Teaching Institution Hayatabad Medical Complex, Peshawar, PAK

**Keywords:** ceruloplasmin, copper metabolism, hepatic dysfunction, urinary copper, wilson's disease

## Abstract

Background

Wilson disease (WD) is a rare autosomal recessive disorder of copper metabolism and an important treatable cause of hepatic dysfunction in young adults. Data from South Asian populations remains limited. Hence, the current study aimed to assess the demographic characteristics, clinical features, and diagnostic profile of patients with WD presenting at a tertiary care hospital in Peshawar, Pakistan.

Methods

This retrospective descriptive study was conducted in the Department of Medicine, Hayatabad Medical Complex, Peshawar, between January 2023 and December 2024. Medical records were systematically reviewed to identify all patients diagnosed with WD during this period. 21 patients with a confirmed WD diagnosis based on the modified Leipzig scoring system (score ≥ 4) and complete diagnostic workup were included. Complete diagnostic data was defined as documented serum ceruloplasmin, 24-hour urinary copper excretion, liver function tests, slit-lamp examination for Kayser-Fleischer (KF) rings, and negative screening for viral and autoimmune hepatitis. Demographic, clinical, and biochemical data were extracted from hospital records and analyzed using SPSS version 25. Post-hoc ethical approval was obtained from the institutional review board.

Results

The mean age at presentation was 23.2 ± 4.8 years (range 18-35), with a male-to-female ratio of 2:1. 15 patients (71.4%) were between 18 and 25 years of age. Jaundice was the predominant symptom, observed in 21 (100%) patients, while hepatomegaly and KF rings were observed in 18 (85.7%) and 12 (57.1%) of patients, respectively. The mean 24-hour urinary copper excretion was 1,450.3 ± 420.7 µg/day, and the mean serum ceruloplasmin level was 8.4 ± 3.2 mg/dL. Four patients (19%) were from two consanguineous families. A positive correlation between urinary copper and total bilirubin levels was observed (r = 0.74, p < 0.001).

Conclusion

WD in this internal medicine cohort primarily affected young adults presenting with hepatic manifestations, most commonly jaundice. Elevated urinary copper excretion and reduced serum ceruloplasmin levels were consistent diagnostic findings. Familial clustering, particularly in consanguineous families, was also observed, indicating a pattern consistent with autosomal recessive inheritance within this population.

## Introduction

Wilson disease (WD), or hepatolenticular degeneration, is a rare autosomal recessive disorder of copper metabolism caused by mutations in the ATP7B gene on chromosome 13q14.3 [1]. These mutations impair biliary copper excretion and incorporation into ceruloplasmin, leading to toxic copper accumulation in the liver, brain, and cornea [2,3]. The resulting oxidative injury produces hepatic, neurological, and psychiatric manifestations, making WD a multisystem disorder with diverse clinical presentations that pose significant diagnostic challenges.

Although the global prevalence is estimated at one in 30,000-100,000 individuals, WD remains one of the few inherited metabolic diseases that is treatable if recognized early [4]. Diagnostic evaluation typically includes serum ceruloplasmin measurement, 24-hour urinary copper excretion, and slit-lamp examination for Kayser-Fleischer (KF) rings [5]. According to the updated American Association for the Study of Liver Diseases (AASLD) Practice Guidance and the European Association for the Study of the Liver (EASL)-European Reference Network (ERN) Clinical Practice Guidelines, a urinary copper excretion exceeding 100 µg/24 hours combined with a Leipzig score of 4 or greater is highly indicative of WD [5,6].

The clinical presentation of WD varies widely from asymptomatic biochemical abnormalities to fulminant hepatic failure or neuropsychiatric symptoms [7]. Hepatic manifestations generally predominate in the second and third decades of life, while neurological involvement, characterized by movement disorders, dysarthria, and cognitive impairment, tends to appear later [8]. Early detection is crucial, as chelation therapy with D-penicillamine, trientine dihydrochloride, or zinc salts can halt disease progression, promote copper mobilization and excretion, and reverse hepatic injury when initiated before the development of decompensated cirrhosis or permanent neurological damage [9]. The dramatic contrast between the devastating natural history of untreated WD and the excellent prognosis achievable with early diagnosis and sustained treatment underscores the critical importance of clinical awareness and systematic diagnostic protocols.

In South Asia, however, awareness of WD remains limited among primary healthcare providers, and diagnosis is often substantially delayed due to overlapping clinical features with viral and autoimmune hepatitis. Up to two-thirds of WD cases may be initially misdiagnosed, with a mean diagnostic delay of up to two years [10]. Pakistan, where consanguineous marriages account for approximately 63% of all unions, may have a substantially higher prevalence of WD and a greater proportion of familial cases compared to populations with lower consanguinity rates, yet systematic epidemiological data remain scarce [11].

To address this gap, the present study describes the demographic characteristics, clinical features, and biochemical profiles of patients with WD presenting at a tertiary care center in Peshawar, Pakistan. The aim is to enhance clinical awareness, support early diagnosis, and contribute to the regional evidence for the global understanding of this treatable inherited disorder.

## Materials and methods

Data were entered into IBM Statistical Package for the Social Sciences (SPSS) version 25.0 (IBM Corp.,USA) for analysis. Data completeness and accuracy were verified through double-entry checking procedures before statistical analysis. Descriptive statistics were used to summarize demographic, clinical, and laboratory variables.

The Shapiro-Wilk test was applied to assess the normality of distribution for continuous variables. Continuous variables were presented as mean ± SD when normally distributed, or as median with interquartile range (IQR) when distribution was non-normal. Categorical variables were presented as frequencies and percentages.

For group comparisons, the Chi-square test or Fisher's exact test was used for categorical data, depending on expected cell frequencies, while the independent-samples t-test was used for normally distributed continuous data, and the Mann-Whitney U test was applied for non-normally distributed continuous data.

Correlations among continuous variables were evaluated using Spearman's rank correlation coefficient due to the small sample size and to avoid assumptions about linear relationships. Given the exploratory nature of this descriptive study and the small sample size, no corrections for multiple comparisons were applied, and correlation findings should be interpreted cautiously as hypothesis-generating rather than confirmatory. Two-tailed p values less than 0.05 were considered statistically significant. The small sample size (n = 21) limits statistical power and the generalizability of findings, particularly for subgroup analyses and correlation estimates, which should be interpreted as preliminary observations requiring validation in larger cohorts.

## Results

A total of 21 (100%) patients diagnosed with WD were included in the analysis. The mean age at presentation was 23.2 ± 4.8 years (range 18-35), and males comprised 66.7% (n = 14) of the cohort, yielding a male-to-female ratio of 2:1. 15 patients (71.4%) were between 18 and 25 years of age. Four patients (19.0%) belonged to two consanguineous families, while the remaining 17 (81.0%) were isolated cases. All patients presented with jaundice as the predominant symptom. Hepatomegaly in 18 (85.7%) patients and splenomegaly in 12 (57.1%) patients were frequent findings, whereas ascites was observed in three (14.3%). KF rings were identified in 12 (57.1%) patients (Table 1).

**Table 1:** Demographic and clinical characteristics of patients with WD (n = 21) KF: Kayser–Fleischer; WD: Wilson disease

Variables	Frequency (n)	Percentage (%)
Gender		
Male	14	66.7
Female	7	33.3
Age group		
18–25 years	15	71.4
26–35 years	6	28.6
Consanguinity		
Consanguineous families	4	19.0
Isolated cases	17	81.0
Clinical features		
Jaundice	21	100.0
Jaundice + Anemia	2	9.5
Hepatomegaly	18	85.7
Splenomegaly	12	57.1
Ascites	3	14.3
KF rings	12	57.1

All patients demonstrated biochemical evidence of hepatic dysfunction. The mean alanine aminotransferase (ALT) was 145.7 ± 78.3 IU/L, aspartate aminotransferase (AST) was 132.4 ± 65.9 IU/L, and total bilirubin was 8.7 ± 4.2 mg/dL. The mean 24-hour urinary copper excretion was 1,450.3 ± 420.7 µg/day, substantially exceeding the normal limit (< 60 µg/day), while mean serum ceruloplasmin was 8.4 ± 3.2 mg/dL, markedly below the normal range (20-40 mg/dL). The mean Leipzig score was 6.2 ± 1.4 (range 4-8), confirming WD in all patients.

All cases tested negative for hepatitis (hepatitis B surface antigen (HBsAg); antibodies to hepatitis C virus (anti-HCV), hepatitis A virus immunoglobulin M (HAV IgM), hepatitis D virus (HDV), hepatitis E virus (HEV)) and autoimmune markers (anti-nuclear antibodies (ANA), anti-smooth muscle antibodies (ASMA), liver-kidney microsomal antibodies type 1 (LKM-1), anti-mitochondrial antibodies (AMA), anti-liver cytosol type 1 (anti-LC1) antibodies, anti-soluble liver antigen (anti-SLA) antibodies, anti-centromere antibodies), effectively excluding other hepatic etiologies (Table 2).

**Table 2:** Biochemical and diagnostic profile of patients with WD Values presented as mean ± SD for continuous variables and N (%) for categorical variables. AST: Aspartate aminotransferase; ALT: Alanine aminotransferase; KF: Kayser–Fleischer; WD: Wilson disease

Variables		Mean ± SD
ALT (IU/L)		145.7 ± 78.3
AST (IU/L)		132.4 ± 65.9
Total Bilirubin (mg/dL)		8.7 ± 4.2
Serum Ceruloplasmin (mg/dL)		8.4 ± 3.2
24-Hr Urinary Copper (µg/day)		1450.3 ± 420.7
Hemoglobin (g/dL)		11.8 ± 2.1
KF rings	Present (2 Points)	12 (57.1)
	Absent (0 Point)	9 (42.9)
Serum Ceruloplasmin	< 10 mg/dL (2 Points)	18 (85.7)
	10–20 mg/dL (1 Point)	3 (14.3)
24-Hr Urinary Copper	> 100 µg/day (2 Points)	21 (100.0)
Total Leipzig Score	4–5 points	6 (28.6)
	6–7 points	11 (52.4)
	8 points	4 (19.0)

Four patients (19.0%) were members of two consanguineous families with first-cousin parental marriages, each family having two affected siblings diagnosed during the study period. The remaining 17 cases (81.0%) were apparently sporadic with no documented family history of WD, though systematic family screening was not uniformly performed or documented. The presence of familial clustering in nearly one-fifth of cases emphasizes the impact of consanguinity on autosomal recessive disease burden in this population (Table 3).

**Table 3:** Family analysis

Family	No. of Affected Siblings	Consanguinity	Age at Diagnosis (years)	Gender
Family A	2	First cousins	22, 24	M, F
Family B	2	First cousins	19, 21	M, M
Sporadic cases	17	Variable	18–35	11M, 7F

A positive correlation was observed between urinary copper excretion and total bilirubin (r = 0.74, p < 0.001), while serum ceruloplasmin showed a moderate negative correlation with urinary copper (r = -0.59, p = 0.006). The Leipzig score also correlated positively with total bilirubin (r = 0.63, p = 0.002) (Table 4).

**Table 4:** Correlation analysis of laboratory parameters Significant correlation at p < 0.05 (two-tailed). Correlation coefficients (r) calculated using Spearman’s rank correlation test. Test statistics represent Spearman’s ρ values. * denotes statistical significance (p < 0.05). KF: Kayser–Fleischer

Variables	r	Test Statistic (ρ)	P-value
Urinary Copper vs Total Bilirubin	0.751	ρ = 0.751	< 0.001*
Urinary Copper vs Ceruloplasmin	–0.679	ρ = –0.679	0.001*
Leipzig Score vs Total Bilirubin	0.624	ρ = 0.624	0.003*
Age vs Urinary Copper	0.389	ρ = 0.389	0.080
KF Rings vs Ceruloplasmin	–0.604	ρ = –0.604	0.004*

Comparison between male and female patients revealed no statistically significant differences in age, biochemical parameters, or Leipzig scores, though males tended to have slightly higher urinary copper and lower ceruloplasmin values (Table 5).

**Table 5:** Comparative analysis by gender Values presented as mean ± SD. Statistical comparisons performed using Mann-Whitney U test for non-parametric data; U statistic values and corresponding two-tailed p-values are presented. P-value < 0.05 (denoted by *) considered statistically significant. No corrections for multiple comparisons were applied.

Variables	Male (n = 14)	Female (n = 7)	Test Statistic (U)	P-value*
Age (years)	21.7 ± 3.5	21.3 ± 4.2	U = 45.5	0.827
Urinary Copper (µg/day)	1361.4 ± 390.2	1346.0 ± 249.5	U = 47.0	0.905
Serum Ceruloplasmin (mg/dL)	7.7 ± 3.2	9.9 ± 3.2	U = 31.0	0.193
Total Bilirubin (mg/dL)	10.0 ± 3.8	9.0 ± 3.6	U = 41.5	0.573
Leipzig Score	6.5 ± 1.2	5.7 ± 1.5	U = 34.0	0.281

## Discussion

The present study adds region-specific evidence on the clinical and biochemical features of WD among young adults in Pakistan. Advances in genetic diagnostic tools have improved WD identification globally, though their application remains limited in resource-constrained settings like Pakistan, where clinical and biochemical diagnosis predominates [1]. This retrospective review of 21 confirmed WD cases represents complete case capture within the Department of Medicine over a two-year period. The mean age at presentation of 23 years corresponds with findings from international registries, where hepatic manifestations typically occur earlier than neurologic or psychiatric forms of the disease, with late-onset presentations also documented [2]. The male predominance observed in 14 (66.7%) patients with a male-to-female ratio of 2:1 is consistent with registry-based data showing gender differences in presentation patterns, with neuropsychiatric subtypes reported as more common among men (67%) compared to women (49%) [12]. These apparent gender differences may partly result from referral patterns and sociocultural factors influencing access to tertiary care rather than true biological variation.

All 21 (100%) patients presented with jaundice, indicating advanced hepatic dysfunction at diagnosis. While our cohort consisted entirely of young adults with chronic presentations, pediatric WD presenting as acute liver failure represents a distinct and severe phenotype documented in meta-analyses, which was not observed in our adult medicine department cohort [3]. Previous studies from Pakistan have reported similar patterns of hepatic presentation in children with fulminant hepatic failure, where WD represents an important etiology [4]. Comparable trends of late presentation with advanced hepatic disease have been reported in Asian cohorts, where significant proportions of patients already had established cirrhosis at diagnosis [13]. Such late recognition underscores persistent diagnostic delay in low- and middle-income settings, where awareness of WD among primary care physicians remains limited. The absence of neurological or psychiatric symptoms in our series reflects the study’s departmental focus on internal medicine admissions and the younger age of the cohort in whom hepatic manifestations typically predominate.

Biochemical findings mirrored established diagnostic patterns, with markedly elevated 24-hour urinary copper excretion and reduced serum ceruloplasmin concentrations. According to updated AASLD practice guidance, urinary copper exceeding 100 µg/24 hours combined with a Leipzig score of 4 or greater is highly indicative of WD [5]. Similarly, EASL clinical practice guidelines emphasize these biochemical parameters as cornerstone diagnostic criteria [6]. The extremely high urinary copper levels recorded in this study, with a mean of approximately 1,450 µg/day, suggest severe hepatic copper overload. Practical guidance from the British Association for the Study of the Liver provides comprehensive investigation and management algorithms that emphasize the diagnostic value of these tests [7]. These findings also align with clinical practice guidelines from India emphasizing the diagnostic utility of biochemical parameters in settings where genetic testing remains inaccessible [8]. The strong positive correlation between urinary copper and total bilirubin, with a correlation coefficient of 0.74, indicates that greater copper burden corresponds with more severe hepatic injury. Understanding the pathophysiology and enduring challenges of WD diagnosis and treatment remains critical despite a century of progress in this field [9].

KF rings were identified in 12 (57.1%) patients, consistent with published literature reporting them in approximately 50-65% of patients with predominantly hepatic presentations [7,8]. The practical guide for investigation emphasizes that the absence of KF rings does not exclude WD diagnosis, particularly in patients presenting before neurological involvement develops [7]. This underscores the importance of comprehensive diagnostic evaluation, including biochemical testing, rather than relying solely on ophthalmological findings, as emphasized in multiple clinical practice guidelines [5,6,8]. The reference handbook on clinical neurology provides comprehensive coverage of WD diagnosis and manifestations across the disease spectrum [14].

Consanguinity was documented in four (19%) patients, representing two families with multiple affected siblings from first-cousin marriages. Population-based genetic studies have shown higher WD prevalence in South Asian and Middle Eastern countries, largely attributed to consanguineous marriage practices and founder mutations. Analysis of global prevalence data from next-generation sequencing demonstrates substantial geographic variation, with higher rates in populations with elevated consanguinity [15]. Updated prevalence studies confirm that WD affects approximately one in 30,000-100,000 individuals globally, with regional variation related to genetic founder effects and consanguinity rates [16]. In Pakistan, where consanguineous marriages account for approximately 63% of all unions, the actual burden of autosomal recessive disorders, including WD, may be substantially higher than in populations with lower consanguinity rates. These findings reinforce the critical need for systematic family screening when WD is diagnosed, as screening of siblings and first-degree relatives can identify presymptomatic affected individuals who may benefit from early treatment before irreversible organ damage occurs.

While our analysis was necessarily limited to hepatic features, WD is recognized as a multisystem disorder. Studies examining comorbidities have documented that common and serious complications occur among adults with WD beyond hepatic and neurological involvement [17]. Comprehensive reviews of other organ involvement describe renal, cardiac, osteoarticular, and endocrine complications [18]. The absence of documented extra-hepatic manifestations in our cohort may reflect early referral for hepatic symptoms before extra-hepatic involvement becomes clinically apparent, the younger age distribution of our patients, or incomplete systematic evaluation for subclinical organ involvement.

Substantial variation in reported prevalence and clinical presentation of WD across different studies likely stems from methodological differences, including diagnostic criteria applied, case ascertainment methods, and population characteristics. Population-based observational studies from France provide important European comparison data, while epidemiological studies from South Korea demonstrate different patterns in East Asian populations [19,20]. Comparison with the Hong Kong Chinese population, where territory-based studies examined epidemiology and natural history between 2000 and 2016, reveals both similarities and differences with our Pakistani cohort, particularly regarding age at presentation and predominance of hepatic manifestations [13].

The pooled global prevalence estimates range between 14 and 34 per million, with substantial geographic variation [15,16]. Such disparities highlight the importance of establishing regional disease registries with standardized diagnostic criteria.

The early age at presentation in our cohort, with 15 (71.4%) patients between 18 and 25 years of age, emphasizes WD as an important differential diagnosis in young adults with unexplained hepatic dysfunction. This age distribution is consistent with the typical presentation of hepatic WD in the second and third decades of life, as documented in the clinical neurology literature [14]. Clinical awareness among primary care physicians and general internists must be enhanced to facilitate earlier recognition before advanced liver disease develops. Earlier detection through screening of high-risk populations, maintaining high clinical suspicion for atypical liver disease in young patients, and ensuring access to specialized diagnostic testing could shift the diagnostic window earlier in the disease course when treatment outcomes are most favorable. Contemporary guidelines emphasize the importance of early diagnosis, as even late-onset WD diagnosed and treated appropriately can have favorable outcomes [2,5-7].

Treatment of WD with chelation therapy can halt disease progression and reverse hepatic injury when initiated before the development of decompensated cirrhosis. Current American practice guidance provides clear recommendations for treatment initiation and monitoring, while European guidelines offer complementary therapeutic algorithms [5,6]. Indian clinical practice guidelines adapted to South Asian contexts provide region-specific treatment recommendations, particularly relevant to Pakistani practice settings [8]. The importance of early diagnosis is further underscored by the excellent prognosis achievable with appropriate lifelong treatment, contrasting sharply with the progressive neurological deterioration and hepatic failure that occur in untreated cases. Despite a century of progress on WD establishing effective therapeutic strategies, enduring challenges in genetics, diagnosis, and treatment implementation persist, particularly in resource-limited settings [9].

This study adds to the limited published data on WD from Pakistan and South Asia. Previous reports from Pakistan examining fulminant hepatic failure have documented WD as an important etiology in young patients, and data on pediatric presentations as acute liver failure provide additional context, though our adult cohort showed chronic rather than fulminant presentations [3,4]. Clinical practice guidelines from India provide a valuable regional context for South Asian populations, but systematic epidemiological data from Pakistan remain scarce [8]. The application of new genetic diagnostic tools remains limited in our setting due to resource constraints, making clinical and biochemical diagnosis particularly important [1]. The current findings support the need for increased clinical awareness, improved diagnostic capacity, and the development of standardized care pathways for WD in Pakistan. Comparison with international registry data from diverse populations including France, South Korea, and Hong Kong demonstrates both universal features of WD and region-specific variations that warrant further investigation [13,19,20].

## Conclusions

In this retrospective cohort of 21 patients with WD presenting to an internal medicine department in Peshawar, most were young adults, with 15 patients (71.4%) aged 18-25 years, and all (100%) exhibited jaundice while 85.7% had hepatomegaly, indicating that diagnosis often occurred only after substantial hepatic dysfunction had developed, as reflected by markedly elevated total bilirubin levels (8.7 ± 4.2 mg/dL), raised transaminases, and exceptionally high urinary copper excretion (mean ~1,450 µg/day). Consistently reduced serum ceruloplasmin and uniform biochemical abnormalities reinforced the diagnostic utility of Leipzig-based criteria in settings where genetic testing is not readily available. The detection of four affected individuals (19%) within two consanguineous families highlights the underlying genetic burden in high-consanguinity populations and underscores the need for family screening. While limited by its small sample size and department-specific case capture, which likely under-represents neurological presentations, this study suggests the importance of earlier clinical suspicion and accessible biochemical testing for young patients with unexplained liver disease, as timely diagnosis may allow treatment initiation before severe and potentially irreversible hepatic injury occurs.
